# Malignancy Associated MicroRNA Expression Changes in Canine Mammary Cancer of Different Malignancies

**DOI:** 10.1155/2014/148597

**Published:** 2014-04-02

**Authors:** Marie-Charlotte von Deetzen, Bernd T. Schmeck, Achim D. Gruber, Robert Klopfleisch

**Affiliations:** ^1^Institute of Veterinary Pathology, Freie Universität Berlin, Robert-von-Ostertag-Straße 15, 14163 Berlin, Germany; ^2^Molecular Pulmonology, German Center for Lung Research, Philipps Universität Marburg, Hans-Meerwein-Straße 2, 35043 Marburg, Germany

## Abstract

MicroRNA has been suspected to be generally involved in carcinogenesis since their first description. A first study supported this assumption for canine mammary tumors when miRNA expression was compared to normal gland. The present study extends these results by comparing the expression of 16 microRNA (miRNA) and 4 small nucleolar RNA (snoRNA) in tumors of different malignancy, for example, adenomas, nonmetastasizing and metastasizing carcinomas as well as lymph node metastases, with each other and with normal mammary gland. All neoplastic tissues differed in their miR-210 expression levels from normal gland. While metastatic cells differed in their expression of mir-29b, miR-101, mir-125a, miR-143, and miR-145 from primary tumors, the comparison of miRNA expression in primary tumors of different malignancy failed to reveal significant differences except for a significant downregulation of mir-125a in metastasizing carcinomas when compared to adenomas.

## 1. **Introduction**

MicroRNA (miRNA) is an evolutionarily conserved, noncoding, but regulatory RNA species of approximately 22 nucleotides in length. It plays a crucial role in various physiological and pathological processes by regulating gene expression posttranscriptionally. miRNA binds to messenger RNA (mRNA) and thereby induces a sequence-depending mRNA degradation or translational repression [1–3]. A deregulation of miRNA is associated with a wide variety of pathologic states including carcinogenesis [4]. Nevertheless, in many cases the specific function of individual miRNA species is still unknown. For instance, miR-10b has been identified as a tumor suppressor which prevents human breast cancer development but also as an oncogene which initiates breast cancer invasion and metastasis [5]. Several miRNA species have been identified to be involved in human breast cancer development including miR-21, miR-145, and miR-210 [6–8]. In veterinary medicine, only a single study is available on miRNA expression in canine mammary tumors. Boggs et al. [7] compared the expression levels of ten miRNA species in malignant mammary tumors and normal canine mammary gland and found a significant deregulation of miR-21, miR-29b, let-7f, miR-15a, and miR-16 in the tumors.

In the present study, we expand these recent findings on the impact of miRNA deregulation on canine mammary tumors by asking for differences in expression levels of four small nucleolar RNA (snoRNA) and 16 canine miRNA with known relevance for human and canine mammary tumor development in tissue samples of normal mammary gland, adenomas, metastasizing, and nonmetastasizing canine mammary carcinomas as well as lymph node metastases.

## 2. Materials and Methods

Mammary gland tissues including regional lymph nodes from 30 dogs submitted to the Department of Veterinary Pathology of the Freie Universität Berlin were included in the study. Clinical data of the dogs included breed, age, and location of tumors. All tissue samples were freshly frozen not later than 30 min after surgical excision and stored at −80°C until further use. Directly adjacent tissue samples were also fixed in 4% neutral buffered formalin, routinely processed stained with haematoxylin and eosin (HE) for histologic examination. Ten simple mammary adenomas, ten simple carcinomas without evidence of tumor cells in the regional lymph node, and ten simple carcinomas with regional lymph node metastases as well as normal mammary gland tissues of ten dogs were selected and macrodissected for this study to assure a tumor content of more than 80%. The fifth sample group contained laser microdissected lymph node metastases from five dogs with simple, metastasizing mammary carcinoma.

The study was approved by the local animal welfare officer. Surgical resection of the tumors was part of the therapy according to the welfare of the animals and to the state of the art of medical science under full anesthesia. The study therefore had no influence on the common diagnostic or therapeutic measures usually applied on animals with mammary gland tumors and inclusion into the study did not induce any additional treatments, pain, or discomfort-inducing manipulations during the entire study.

Total RNA was extracted as previously described [9, 10]. The lymph node metastases were microdissected from cryostat sections by laser capture microdissection as previously described [11]. All miRNA and snoRNA species were elongated prior to cDNA synthesis by universal poly-A-tailing and amplified by quantitative reverse transcription PCR as previously described [9]. Housekeeping genes were selected from the complete set of genes analyzed using both geNorm (version 3.4) and NormFinder (version 20) algorithms [12, 13]. The most stably expressed RNA species were determined by calculating the gene expression stability measure value (*M*) using the geNorm tool as previously described [12]. Stepwise exclusion of the least stable genes identified miR-155, let-7f, and miR-181b as the three most stably expressed genes. In a second approach NormFinder analysis was performed [13] and identified miR-181b, U44, and U48 as the three most stable reference genes. Considering both the results of the geNorm analysis and the NormFinder algorithm miR-181b, miR-155, and U44 were used for data normalization. Consequently, relative expression levels of the miRNA and snoRNA species were determined using the 2^−ΔΔCt^ method [14]. Due to the different efficiencies of the PCR assays, the actual efficiencies were used as the base of the exponential amplification function for calculation. Statistical significance of differences in miRNA and snoRNA expression levels was evaluated using the IBM SPSS Statistic 20 software. The results in the different tissue groups were statistically compared using the parametric ANOVA analysis. A *P* ≤ 0.05 was considered statistically significant.

## 3. **Results**

**Figure 1:**
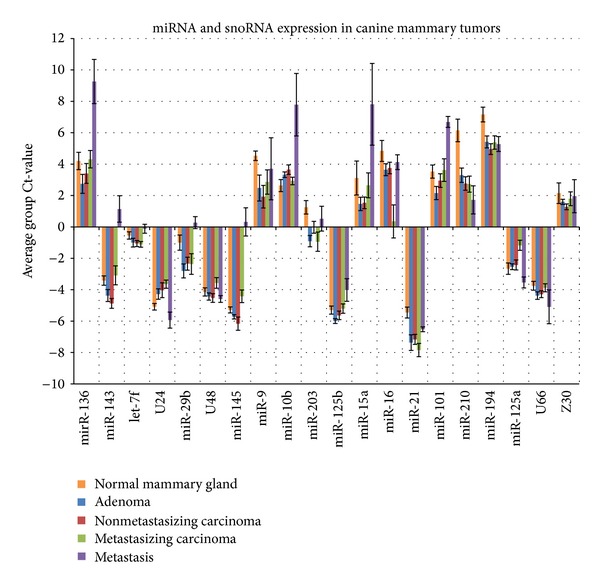
miRNA and snoRNA expression levels in normal gland, adenoma, nonmetastasizing carcinoma, metastasizing carcinoma, and lymph node metastasis. Ct-values are average values of expression levels of the tissue groups normalized to three housekeeping genes.

Comparison of the expression levels of 16 miRNA and four snoRNA leads to the identification of statistically different expression levels of nine miRNA and one snoRNA in 27 of the 400 comparisons. Of these, microdissected tumor metastases differed most often from other tissue types. miR-101, miR-143, and miR-145 were differently expressed in metastases when compared to all other tissues. miR-29b differed in its expression level between metastases and all tumor tissue samples except normal mammary gland. miR-125a expression only differed between metastases and metastasizing carcinomas primary sites (Table 1, Figure 1). The comparison of the primary tumors of different malignancies identified a difference in miR-125a expression between metastasizing carcinoma and adenoma as the only significant difference (Table 1, Figure 1). All other differences in expression were restricted to comparisons between normal mammary gland and neoplastic tissues. miR-21, miR-143, miR-194, miR-203, miR-210, and the snoRNA U24 differed in their expression levels between normal gland and neoplastic tissues (Table 1, Figure 1). Of note, only miR-125a and the snoRNA U24 showed a decreased expression in the primary tumors when compared to normal gland or adenomas while all other significantly regulated miRNA had an upregulation in benign and malignant primary tumors (Table 1). This was in contrast to the observation in metastases which, except for miR-125a and miR-210, had a general downregulation of miRNA when compared to all other tissues (Table 1).

miR-210 was the only miRNA which allowed the differentiation of normal mammary gland from all neoplastic tissues. In addition, miR-210 was the only miRNA with a continuous significant increase in expression levels between normal mammary gland and all tumor groups and the metastases with expression differences of 7.01-fold between adenomas and normal gland, 10.41-fold between nonmetastasizing carcinomas and normal gland, 10.72-fold between metastasizing carcinomas and normal gland, and 19.63-fold between metastases and normal gland (Table 1).

U24 was the only snoRNA with a significant difference in expression. The expression difference was 0.38-fold between the metastasizing carcinoma and the normal mammary gland but was not significantly altered in any other comparison. miR-9, miR-10b, miR-15a, miR-16, miR-125b, miR-136, and let-7f as well as the snoRNA U66, Z30, and U48 had no significant difference in expression between any of the tissues analyzed.

## 4. Discussion

The aim of the present study was the identification of malignancy associated miRNA expression to discover potential malignancy marker and to further elucidate aspects of the molecular carcinogenesis and gene expression associated with metastatic behavior of canine mammary tumors [15]. To this end, the expression of 16 miRNA and four snoRNA was compared between normal mammary gland, adenomas, carcinomas, and metastases. Only nine of the miRNA species and one snoRNA tested were significantly differently expressed between the diverse tissues analyzed.

Interestingly, miR-210 was significantly overexpressed in all neoplastic tissues when compared to normal mammary gland. Previous work described miR-210 as a “hypoxamir” which is upregulated in hypoxic tissues by HIF-1 action and mediates a metabolic adaptation to anaerobic conditions [16]. The continuous upregulation of miR-210 over the course of malignant progression may therefore be caused by an increasing hypoxia in the developing tumor mass. miR-210 overexpression has also been associated with the formation of capillary-like structures [17]. It can thus be hypothesized that miR-210 may indirectly promote metastasis by triggering angiogenesis in neighboring cells [18]. Another four miRNA, miR-21, miR-143, miR-194, and miR-203, were significantly increased in at least one mammary tumor group compared to normal mammary gland and therefore matched the definition of an oncogenic miRNA. While this disease association is new for miR-143 and miR-194, a similar oncogenic function has been suggested for miR-203 [19] in mammary cancer and for miR-21 in several other tumors including human breast cancer but has to be determined yet [20].

The general lack of differences in miRNA expression between primary tumors at different stages of malignancy was intriguing. Only miR-125a was significantly differently expressed between metastasizing carcinoma and adenoma. This was in contrast to the numerous miRNA differentially expressed in metastatic tumor cells in the lymph nodes. This difference may be caused by the different preparation of metastatic tumor cells by laser microdissection technology due to the otherwise high contamination of metastases tissue samples by lymphatic cells. The macrodissected samples of normal gland and the primary tumors in contrast also contained, although at a minimal portion, cells of the tumor stroma. These cells may have contributed to the recorded downregulation of the four miRNA in microdissected metastases when compared to macrodissected primary tumors and normal mammary gland, respectively.

## 5. Conclusions

In conclusion, canine mammary tumors at different stages of malignancy significantly differed from normal gland in the expression of seven miRNA and one snoRNA species. miRNA and snoRNA expression however failed in most cases to discriminate primary tumors at different stages of malignancy.

## Figures and Tables

**Table 1:** miRNA and snoRNA expression differences between mammary tissues at different stages of malignancy and normal gland.

	Normal	Adenoma	Nonmetastasizing carcinoma	Metastasizing carcinoma	Metastasis	Regulation
Normal		miR-203 (4.37) miR-210 (7.01)	miR-143 (2.70) miR-21 (3.18) miR-210 (10.41) miR-194 (4.44)	miR-21 (5.05) miR-210 (10.72)	miR-210 (19.63)	Up
				U24 (0.38)	miR-143 (0.04), miR-145 (0.02), miR-101 (0.12)	Down

Adenoma						Up
				miR-125a (0.40)	miR-143 (0.02), miR-145 (0.02), miR-101 (0.05), miR-29b (0.14)	Down

Nonmetastasizing carcinoma						Up
					miR-143 (0.02), miR-145 (0.01), miR-101 (0.08), miR-29b (0.18)	Down

Metastasizing carcinoma					miR-125a (4.91)	Up
					miR-143 (0.06), miR-145 (0.04), miR-101 (0.13), miR-29b (0.18)	Down

Metastasis						Up
						Down

In brackets (): fold change in expression difference.
